# The Patriarchs of Pinner

**Published:** 1856-10

**Authors:** John Webster


### ORIGINAL COMMUNICATIONS.

### THE PATRIARCHS OF PINNER, MIDDLESEX.

By JOHN WEBSTER, M.D., F.R.C.P., F.R.S.

In previous numbers of the Journal of Public Health I briefly communicated some curious grave-stone statistics, personally collected from different cemeteries in the vicinity of London, in East Anglia, and throughout several central counties of Scotland, my chief object in instituting these researches being to ascertain the real or the comparative salubrity of particular districts. However, when commenting upon the facts so obtained, I stated that the inferences drawn should not be received as *absolutely* correct, but as approximations only to a general truth. As further illustrating the question there discussed, I now refer to a locality I have recently visited, where very old age seems exceedingly common amongst the inhabitants. The place here alluded to is Pinner, situate about thirteen miles north-west of London, and nearly three beyond Harrow-on-the-Hill. This part of Middlesex occupies high lying ground, adjoining a feeder of the river Colne, and constitutes almost entirely a grass-growing or grazing district. Its surface being undulating, and ornamented by villas and many fine trees, with truly luxuriant plantations, the environs of Pinner are very beautiful. The township itself, or village, is small, looks rural and primitive, but pretty; whilst most of the houses or humble tenements appear to have undergone few changes since they were originally constructed. As evidence of this, the principal inn, where weary travellers can refresh themselves, may be confidently pointed out, as it exhibits the same outward aspect as it did in 1711, when first built; that date being visible on the exterior of this very unpalatial looking hostelry, which consists chiefly of lath and plaster, with an overlapping upper story, like many antiquated metropolitan dwellings erected at a parallel period. The ancient parish church, founded in 1322, should be also named, since it has continuously served for celebrating divine worship during upwards of five centuries.

Within the gently sloping, yet somewhat picturesque, burying-ground,—although by no means of large extent, or marked by numerous monuments,—the very great ages inscribed on many of such of its death memorials as still exist, are unmistakeably eloquent of longevity, when compared with similar records noticed elsewhere. Thus few graves formed the resting places of very young persons. Several were of persons between 65 and 80 years; whilst, from that advanced period of life to beyond 100, not less than thirty-six instances were noted, comprising eighteen of each sex, which may be thus classified. Of men, three died at 80; three at 82; one at 84; one at 85; one at 86; three at 87; two at 88; one at 90; one at 92; one at 94; and one man had actually attained 118 years! Amongst the women, three died at 80; one at 81; four at 84; two at 85; two at 86; two at 89; one at 92; one at 94; one at 100; and another was a centenarian, being 102 years old at her death. Respecting this last-named woman—Betty Evans—it should be mentioned, that she had a sister, buried at Watford, who lived to 104; that she was born at Ruislip (an adjacent hamlet, whose cemetery also contains centenarians), died in 1853, and was buried only a few years after her elder relative. The male patriarch reported to have seen *a hundred and eighteen* winters, was named William Skenelsby, and departed life on the 7th of November, 1775; but regarding his birth-place no information can be derived from his modest grave memorial. To indicate still more conclusively the unusual longevity frequently characterising modern dwellers in this neighbourhood, it becomes important to add that the present clergyman has performed funeral services over the graves of two of his centenarian parishioners. Besides this peculiarity, from the 1st of January, 1856, to August 24th, when I perambulated Pinner churchyard, to verify the mortuary details just chronicled, the same gentleman informed me that five persons, who at death were 82 years and upwards, had been buried therein by him, of whom three were men, aged 82, 86, and 94 respectively, with two women, one being 88, and the other 85; the funeral of the last took place in the afternoon of my visit.

Taking all the facts above enumerated into consideration, it may be fairly inferred that Pinner is a healthy residence, and occupies an elevated position in the scale of longevity, speaking comparatively. No district alluded to in my former reports, published in the JOURNAL OF PUBLIC HEALTH, supplies so large a proportion of very old people as this, which hence becomes an instructive and singular example of many long-lived persons belonging to one locality. It is only necessary to add, that the annual burials in this country cemetery range from eighteen to twenty, amongst a population of not more than 1310, according to the census last published.

24, Brook Street, Grosvenor Square, September 1856.

